# Serum Metabonomic Study of Patients With Acute Coronary Syndrome Using Ultra-Performance Liquid Chromatography Orbitrap Mass Spectrometer

**DOI:** 10.3389/fcvm.2021.637621

**Published:** 2021-02-26

**Authors:** Lei Song, Zhongxiao Zhang, Zhaohui Qiu, Tingbo Jiang

**Affiliations:** ^1^The First Affiliated Hospital of Soochow University, Suzhou, China; ^2^Tongren Hospital, Shanghai Jiao Tong University School of Medicine, Shanghai, China; ^3^Hongqiao International Institute of Medicine, Tongren Hospital, Shanghai Jiao Tong University School of Medicine, Shanghai, China

**Keywords:** acute coronary syndrome, metabonomic, serum, biomarkers, UPLC-Orbitrap/MS

## Abstract

Acute coronary syndrome (ACS) can cause arrhythmia, heart failure, and even sudden death. Our aim in this study was to identify potential metabolic biomarkers in patients with ACS. The serum metabonomics approach based on ultra-performance liquid chromatography (UPLC)/Orbitrap mass spectrometer (MS) was used to analyze the serum samples from 45 patients with ACS and 29 healthy controls. Multivariate statistical analysis was used to screen for ACS biomarkers. In total, 69 biomarkers were identified to be enriched in 19 metabolic pathways; 43 biomarkers were significantly up-regulated, while 26 biomarkers were significantly down-regulated in the ACS group. The main classes were lyso-sphingolipid (SM), cinnamic acids, cholines, and primary amides. Receiver operating characteristic (ROC) curve analysis showed that lysoPC(20:4(8Z,11Z,14Z,17Z)/0:0) (ROC area under the curve, AUC = 0.936), SM(d18:0/16:0) (ROC AUC = 0.932), and SM(d18:1/14:0) (ROC AUC = 0.923) had a high ACS diagnostic ability. The AUC value of the diagnostic model constructed using these combined biomarkers was 0.96. Therefore, these biomarkers may improve the diagnostic efficacy of ACS. The findings of this study also implied that glycerophospholipid metabolism; the biosynthesis of unsaturated fatty acids; linoleic acid metabolism; and valine, leucine, and isoleucine biosynthesis played important roles in ACS. Network analysis by ingenuity pathway analysis (IPA) showed these biomarkers were correlated to the cardiac hypertrophy signaling pathway, ERK/MAPK signaling pathway, NF-kappa B signaling pathway, nitric oxide (NO) signaling pathway in cardiovascular system, and TLR-signaling pathway. These findings will help to improve the ability of accurate diagnosis and intervention of ACS.

## Introduction

Acute coronary syndrome (ACS) is an acute thrombosis that occurs after the rupture of unstable plaque characterized by acute myocardial ischemia (1, 2). ACS is often accompanied by atherosclerotic plaque rupture and thrombosis, related to severe cases (3), and can cause arrhythmia, heart failure, and even sudden death (4). If timely detection and intervention can be performed, its mortality and complications can be greatly reduced, and the prognosis of patients can be effectively improved (5). At present, the traditional biomarkers used in risk assessment cannot predict the occurrence of acute cardiovascular events (6, 7); therefore, this approach is limited to determining the prognosis. The commonly used diagnostic methods, such as troponin, cannot diagnose patients with early ACS (8). In addition, approximately 50% of patients do not have typical electrocardiogram (ECG) manifestations of ACS (9). About 25% of patients do not have the typical characteristics of clinical ACS in an early stage, which makes the treatment strategy choice more difficult (10). For these patients, timely detection and diagnosis have become an urgent problem that needs to be solved. Therefore, we urgently need a more targeted indicator (11).

Metabonomics is a newly developed discipline that can be used for qualitative and quantitative analysis of all low molecular weight metabolites of organisms or cells under physiological or pathological conditions (12). It is widely used in the diagnosis of a variety of diseases (13), such as neuropsychiatric diseases (14), liver fibrosis (15), and liver cancer (16). Metabolomics has been used to screen specific metabolites for risk assessment and prediction in cardiovascular diseases, such as ACS and hyperlipidemia (17, 18).

In this study, we used liquid chromatography-mass spectrometry (LC-MS-MS) to analyze the serum samples of patients with ACS at different pathological stages and to identify the specific metabolites that are used to diagnose ACS patients. It is helpful to provide a reference for the study of the pathogenesis of coronary heart disease (CHD).

## Methods

### Patient Recruitment

This study included patients who underwent coronary angiography or coronary computed tomography angiography (CTA) in the Department of Cardiology of Shanghai Tongren Hospital from January 2018 to May 2020. We recruited 45 cases of acute coronary syndrome group (ACS group), including 12 cases of acute non-ST segment elevation myocardial infarction (NSTEMI), 25 cases of unstable angina pectoris (UA), eight cases of acute ST segment elevation myocardial infarction (STEMI), and 29 healthy subjects (control group). After admission, all patients provided their personal information, medical history, genetic history, and family medical history, as well as their consent for data collection. The study protocol was approved by the Ethical Review Committee of Shanghai Tongren Hospital (Shanghai, China). Written informed consent was obtained from all the participants before sample collection.

*Diagnostic criteria: UA:* Angina pectoris changes not only with the frequency of attacks but also with the onset of pain, since when it is increased, the onset time is longer than the original. Angina attacks occur during rest. In the previous month, angina pectoris had occurred due to physical exercise; a previous abnormal coronary angiography and positive exercise test results had been obtained. ECG showed that two similar leads showed dynamic ST segment or T wave changes, but the myocardial necrosis markers were normal. *NSTEMI:* With ACS symptoms and elevated CTN, the ECG ST segment did not rise. *STEMI:* The criteria are symptoms of myocardial ischemia, new ischemic ECG changes, development of pathological Q waves, and imaging evidence of new loss of viable myocardium or new regional wall motion abnormality in a pattern consistent with an ischemic etiology.

*Case group:* The inclusion criteria are the following: patients who met the diagnostic criteria for ACS and underwent coronary angiography after admission. NSTEMI, UA, and STEMI were also referred to as ACS. Coronary angiography was required to confirm the diagnosis. Exclusion criteria are the following: patients with aortic dissection, pulmonary embolism, malignant tumor, autoimmune disease, severe infectious disease, trauma, recent surgery, severe heart failure, and severe renal insufficiency. We also excluded patients with myocarditis, pericarditis, and other cardiomyopathy.

*Control group:* People in the control group were those with no definite abnormality on coronary CTA or coronary angiography. Exclusion criteria were as follows: (1) malignant arrhythmia, valvular heart disease, aortic dissection, pulmonary embolism, and other diseases; (2) severe liver failure, renal insufficiency (cirrhosis, hepatitis, renal failure, uremia, among others); (3) hematological diseases (multiple myeloma, hemolytic anemia, aplastic anemia, among others); (4) infectious diseases, malignant tumors, autoimmune diseases, serious electrolyte disorders, among others; (5) recent bleeding or infarction disease (cerebral hemorrhage, cerebral infarction, pulmonary embolism, and another organ embolism) had occurred; and (6) diabetes, hypertension, and hypercholesterolemia.

### Sample Collection and Preparation

The fasting blood sample was taken on the first day after admission for the patients who met the inclusion criteria of this study. Venous blood was drawn through the elbow vein before coronary angiography. Two tubes of venous blood (1 ml each) were taken from each patient at the same time. The blood was centrifuged at 3,500 rpm for 10 min at 4°C. The supernatant was transferred into a 1.5 ml centrifuge tube as the serum sample: one tube of serum was used for LC-MS detection, and the other blood sample was used for the detection of clinical biochemical indexes, including triglyceride (TG), high-density lipoprotein (HDL), low-density lipoprotein (LDL), apolipoprotein A1 (APOA1), creatine kinase isoenzyme MB (CKMB), cardiac troponin I (cTNI), urea nitrogen (UN), and epidermal growth factor receptor (eGFR). Each separated frozen serum sample was thawed at room temperature. Approximately 100 μl serum was added to 300 μl of a methanol solution (containing 5 μg/ml L-2-chloro-phenylalanine as an internal standard) and vortexed for 2 min. Centrifugation was performed at 13,000 rpm at 4°C for 10 min, and a 200-μl supernatant was obtained. The same volume of serum was taken from all samples and mixed evenly to prepare the QC (quality control) samples.

### UPLC-Orbitrap/MS

The UPLC system was coupled to an Orbitrap/MS (Waters Corp., Milford, MA, USA) equipped with an electrospray ionization source and operated in either positive or negative ionization mode using a mass resolution of 70,000 at an *m*/*z* of 200. Data-dependent (dd-MS2, TopN = 10) MS/MS mode with a full scan mass resolution of 17,500 at an *m*/*z* of 200 was used. The scan range was 100–1,500. The chromatographic conditions were as follows: injection volume, 2 μl; column temperature, 25°C; flow rate, 0.35 ml/min; and mobile phase, liquid aqueous solution (0.1% formic acid), and liquid b-acetonitrile (0.1% formic acid). The optimized chromatographic gradient was as follows: 0–2 min, 5% in liquid B; 2–10 min, 5–95% in liquid B; 10–15 min, 95% in liquid B; 15–18 min, 5% in liquid B. Data were acquired in centroid mode using Thermo Excalibur 2.2 software (Thermo Fisher Scientific, MA, USA).

### Data Processing and Statistical Analysis

Data were acquired using Thermo Xcalibur 2.2 software (Thermo Scientific, San Jose, USA). Peak alignment and extraction were performed using Compound Discoverer software (Thermo Fisher Scientific). Then, we obtained a data table containing the information regarding the retention time, *m*/*z*, and peak area. Next, the data were imported into Simca-P software version 13.0 (Umetrics, Umea, Sweden) for principal components analysis (PCA) and partial least squares discrimination analysis (PLSDA). The ion peaks were normalized and Pareto-scaled. Unsupervised PCA analysis was used to assess the overall trend of segregation between these samples. A supervised PLSDA analysis model was used to screen for significantly different metabolites between the ACS and control groups. According to PLSDA model, the variables with variable importance in the projection (VIP) value >1.0 were selected, and a two-tailed Student's *t*-test was used to determine *p* performed by SPSS Statistics 18.0.0, and *p* < 0.05 was considered as statistically significant. Bonferroni correction was used for multiple testing adjustment. In order to identify these potential biomarkers, the accurate ion mass was input into the human metabolome database (HMDB), Metlin, MoNA, and massbank databases to match the exact molecular weight, and MS1/MS2 fragment ions were automatically searched. Finally, in order to confirm the structure of the compound, we used our internal standard metabolite library, matching the exact mass, fragment ion mass, and retention time. Pathway enrichment analysis was performed using the Kyoto Encyclopedia of Genes and Genomes (KEGG) database and metaboanalyst (https://www.metaboanalyst.ca/). The diagnostic efficacy value of biomarkers was investigated using ROC curve analysis by using metaboanalyst. The metabolite interaction network analysis was performed using the IPA (ingenuity pathway analysis) online database.

## Results

### Clinical Characteristics

There was no significant difference in age, sex, or body mass index (BMI) between the two groups (*p* > 0.05). The levels of cTNI, APOA1, and CKMB in ACS patients were higher than those in healthy subjects (*p* < 0.05) (Table 1). There were no significant differences in other indexes.

**Table 1:** Baseline characteristics of healthy and ACS patients.

**ID**	**Control**		**ACS**		***p***
		**UA**	**NSTEMI**	**STEMI**	
Number	29	25	12	8	
Gender (ratio)	16/13		18/27		
Age	63.3 ± 7.8		63.7 ± 8.0		0.67
APOA1 (g/L)	1.40 ± 1.45		4.40 ± 5.45		0.04
CKMB (U/L)	0.37 ± 18		25 ± 318		0.01
cTNI (μg/L)	0.02 ± 0.13		0.5 ± 36		0.01
UN (mmol/L)	5.4 ± 0.9		5.3 ± 0.8		0.44
Cr (μmol/L)	67 ± 14.8		68 ± 20.8		0.73
eGFR (CKD-EPI) ml/min/1.73 m^2^	102 ± 23.1		103 ± 29.4		0.84
UA (μmol/L)	360 ± 74.2		365 ± 94.6		0.81
TG (mmol/L)	1 ± 0.6		1 ± 0.5		0.13
CHOL (mmol/L)	4 ± 0.7		4 ± 1.0		0.92
HDL (mmol/L)	1 ± 0.3		1 ± 0.3		0.06
LDL (mmol/L)	3 ± 0.8		3 ± 1.0		0.75
APOB (g/L)	1 ± 0.2		1 ± 0.2		0.20
APOE (mg/dl)	3 ± 0.8		3 ± 1.0		0.35
HCY (μmol/L)	13 ± 4.0		13 ± 6.0		0.80
hsCRP (mg/L)	2 ± 0.8		3 ± 1.0		0.22
k (mmol/L)	4 ± 0.3		4 ± 0.4		0.65
Na (mmol/L)	142 ± 1.9		141 ± 2.2		0.07
MYO (ng/ml)	34 ± 1.4		45 ± 1.8		0.28

*Values are mean ± SD. APOA1, apolipoprotein A1; CKMB, creatine kinase isoenzyme MB; cTNI, cardiac troponin I; UN, urea nitrogen; Cr, creatinine; eGFR, epidermal growth factor receptor; UA, unstable angina; TG, triglyceride; CHOL, total cholesterol; HDL, high-density lipoprotein; LDL, low-density lipoprotein; APOB, apolipoprotein B; APOE, apolipoprotein E; hsCRP, high sensitivity C-reactive protein; MYO, myoglobin*.

### Screening and Identification of Biomarkers

**Figure 1:**
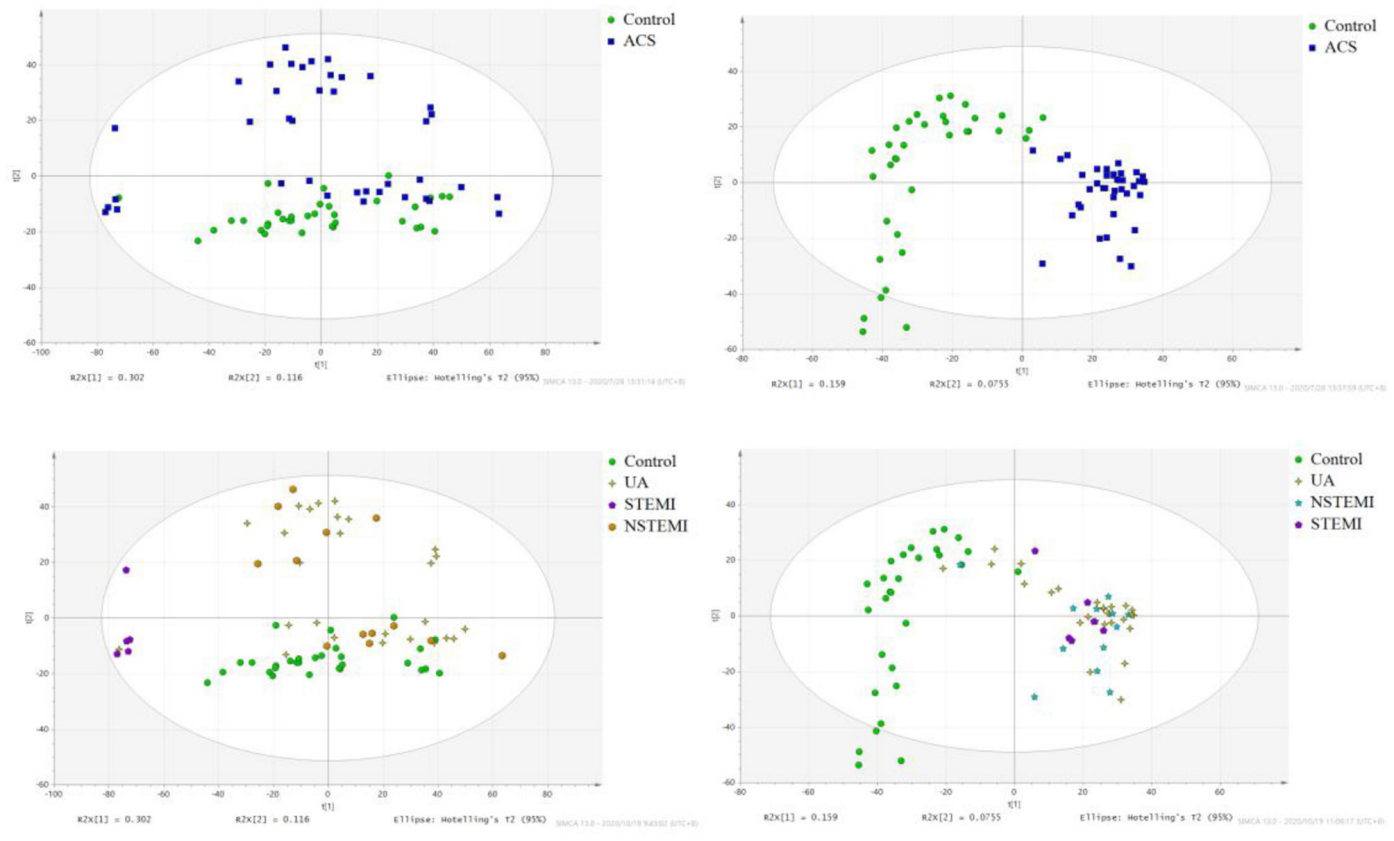
The score plot of PCA analysis. (•) control; (■) ACS group. NSTEMI, non-ST segment elevation myocardial infarction; 25 cases of UA, unstable angina pectoris; STEMI, acute ST segment elevation myocardial infarction; control, healthy subjects.

**Figure 2:**
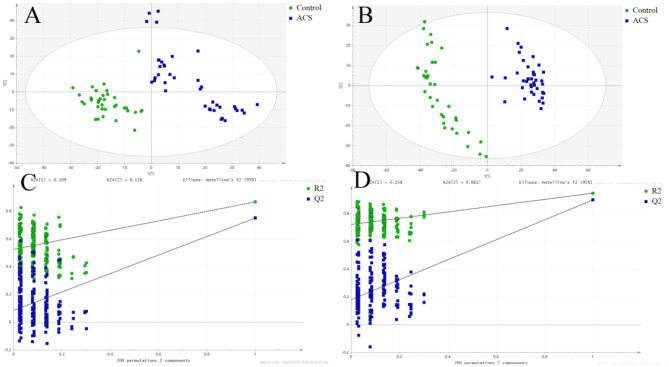
The score plot of the PLSDA model. (•) control; (■) ACS group. **(A)** POS, the score plot of the PLSDA model in the positive mode; **(B)** the score plot of the PLSDA model in the negative mode; **(C)** permutation test of the PLSDA model in the positive mode; **(D)** permutation test of the PLSDA model in the negative mode.

**Figure 3:**
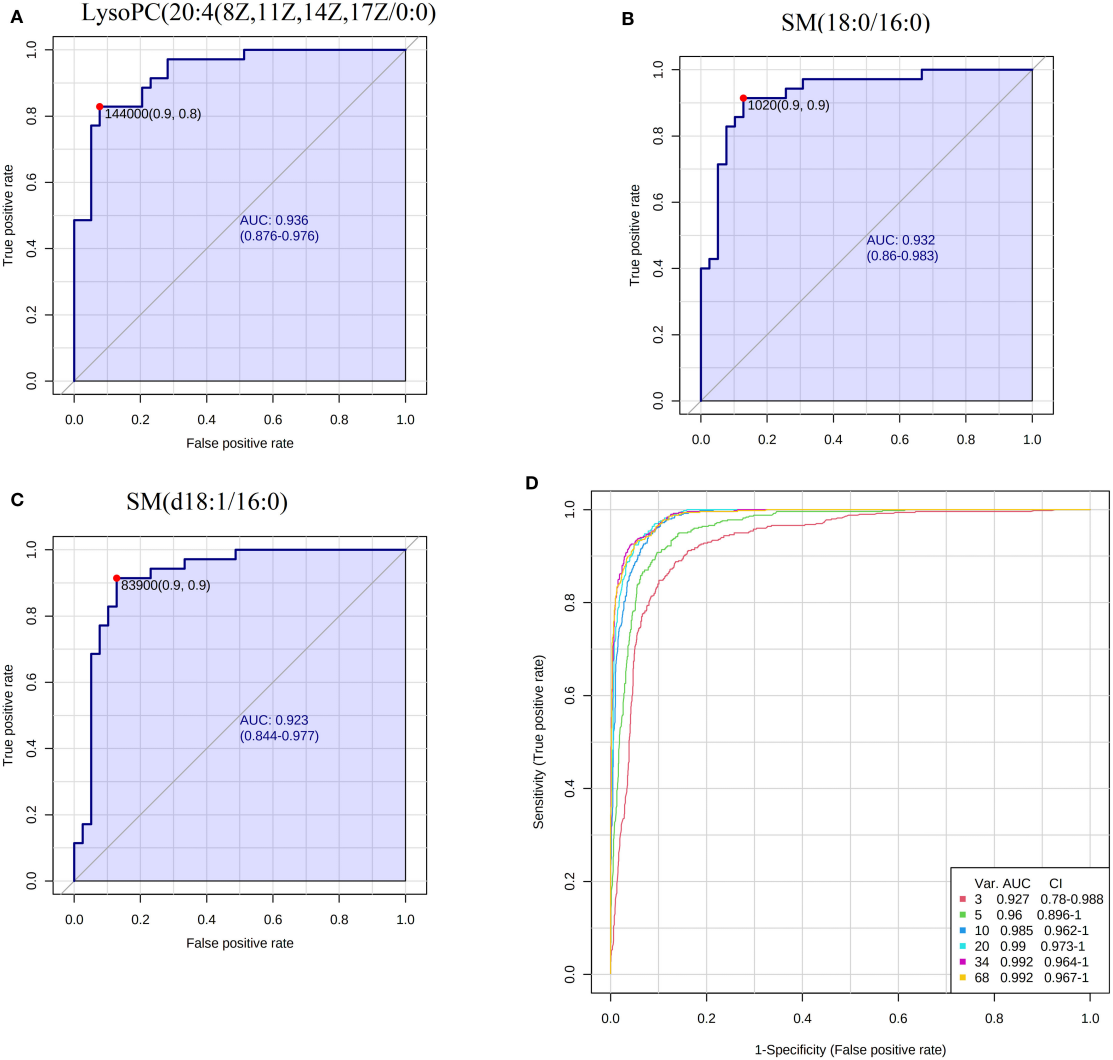
ROC analysis of biomarkers. **(A)** The AUC of LysoPC20:4(8Z,11Z,14Z,17Z/0:0); **(B)** the AUC of SM(18:0/16:0); **(C)** the AUC of SM(d18:1/16:0); **(D)** the combined ROC model of the biomarkers and cTNI.

**Figure 4:**
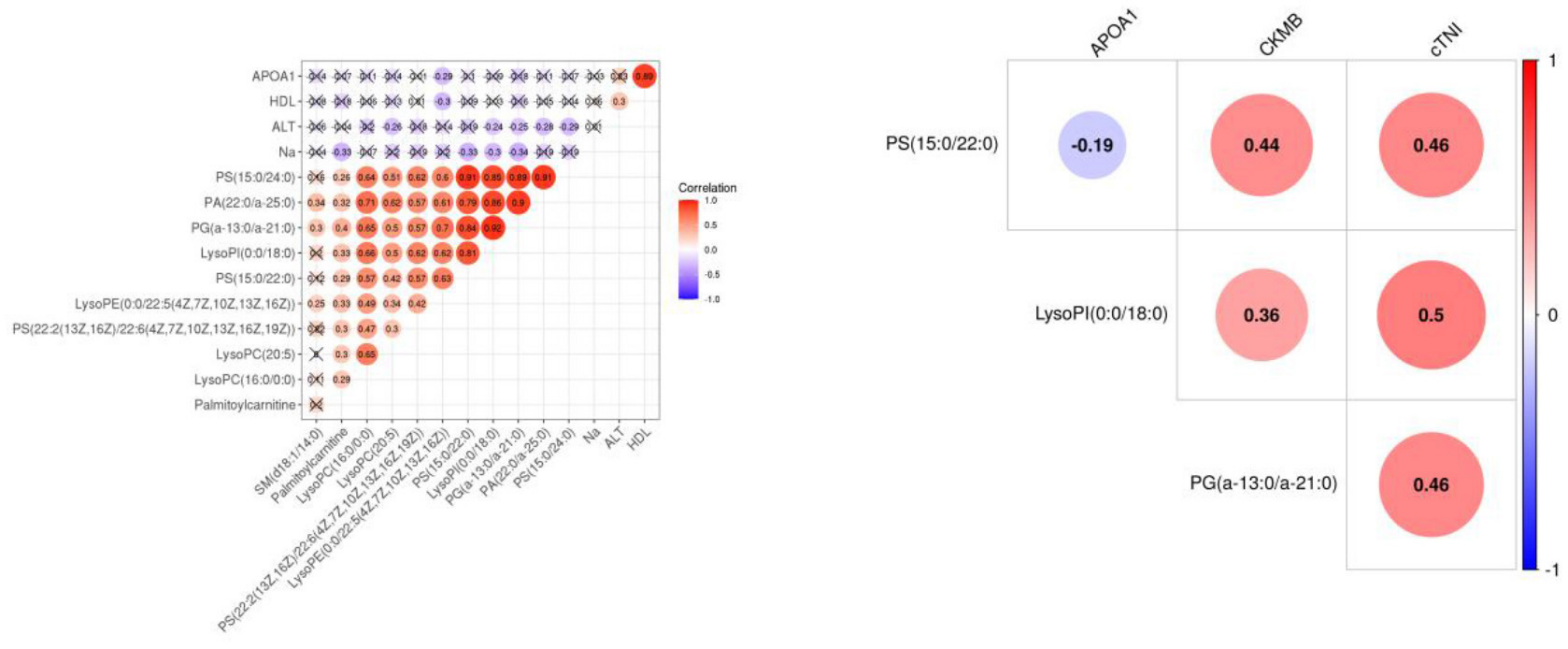
The heat map of the correlation between differential metabolites and clinical indicators APOA1, CKMB, and cTNI. Red represents a positive correlation, and blue represents a negative correlation. The number in the circle represents the correlation coefficient. APOA1, apolipoprotein A1; CKMB, creatine kinase isoenzyme MB; cTNI, cardiac troponin I.

The results of PCA analysis (Figure 1) showed the trend of intra group aggregation and inter-group segregation in the control and ACS groups. For biomarker screening, the PLS-DA model was established. As shown in Figure 2, the ACS group results was far from those of the control group, indicating that there were differences in metabolites between the two groups. A total of 46 differentially expressed metabolites with a VIP >1 and a *p* < 0.05 were identified. A total of 35 metabolites were increased, 11 were decreased, as shown in Supplementary Table 1. The ROC analysis showed that LysoPC(20:4(8Z,11Z,14Z,17Z)/0:0), SM(d18:0/16:0), and SM(d18:1/14:0) showed a high ACS diagnostic ability with area under the curve (AUC) values of 0.936, 0.932, and 0.923, respectively. The AUC of cTNI (0.759) was lower than that of cTNI, and the AUC value of the diagnostic model constructed using the combined metabolic biomarkers with cTNI was 0.96, as shown in Figure 3. This indicated that the combined diagnosis model had a stronger diagnostic ability and that it could be used as a reference for clinical diagnosis. The correlation analysis between metabolites and cTNI, APOA1, and CKMB showed that there was a significant positive correlation between cTNI and PS(15:0/22:0), lysoPI(0:0/18:0), and PG(a-13:0/a-21:0), with correlation coefficients of 0.46, 0.5, and 0.46, respectively, as shown in Figure 4.

### Pathway Analysis of the Biomarkers of ACS

**Figure 5:**
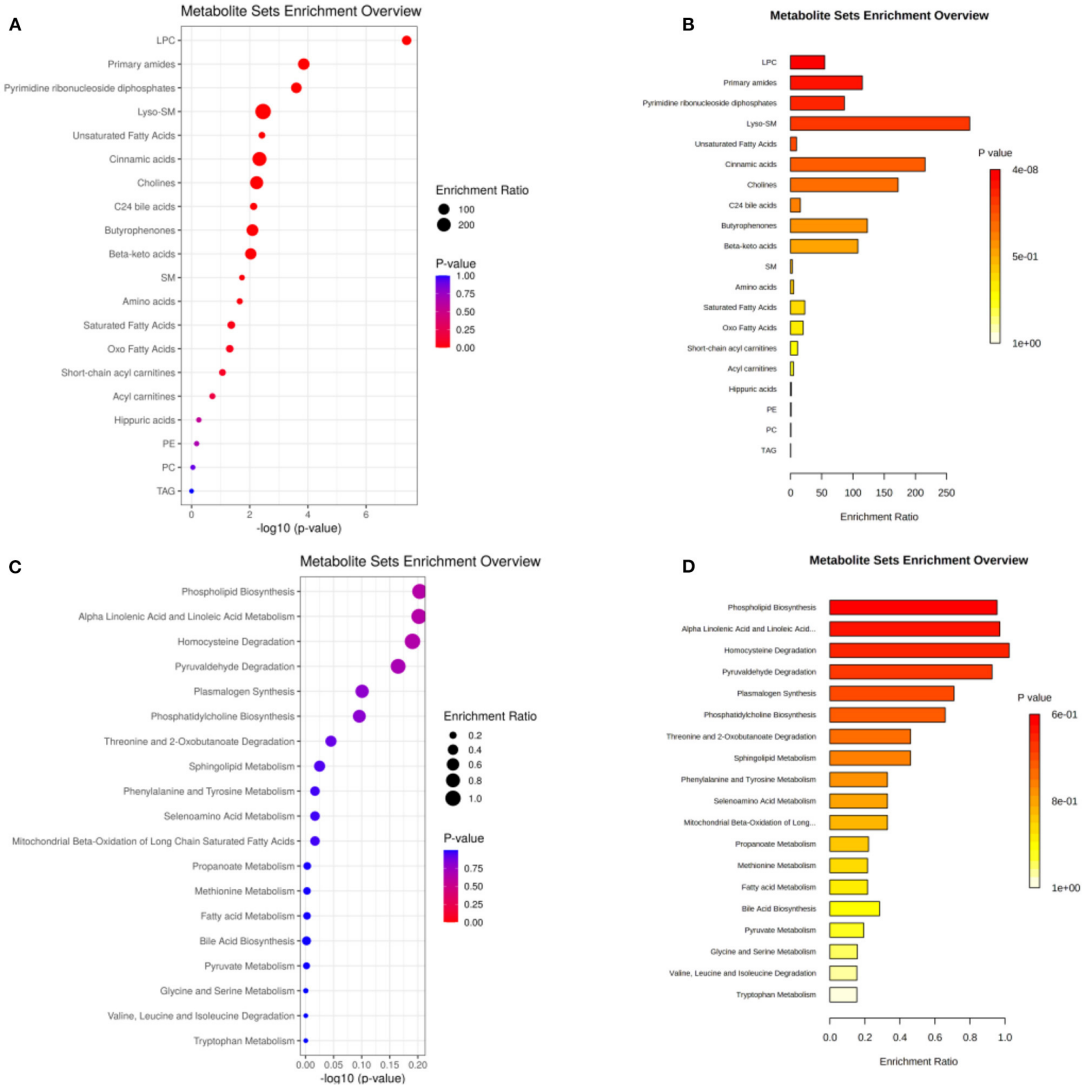
**(A,B)** The main class of the changed metabolites. **(C,D)** Pathway enrichment of the biomarkers. The size of the dots represents the enrichment ratio. The dot color represents the *p-*value, red represents the significant difference, and blue represents the insignificant difference.

**Figure 6:**
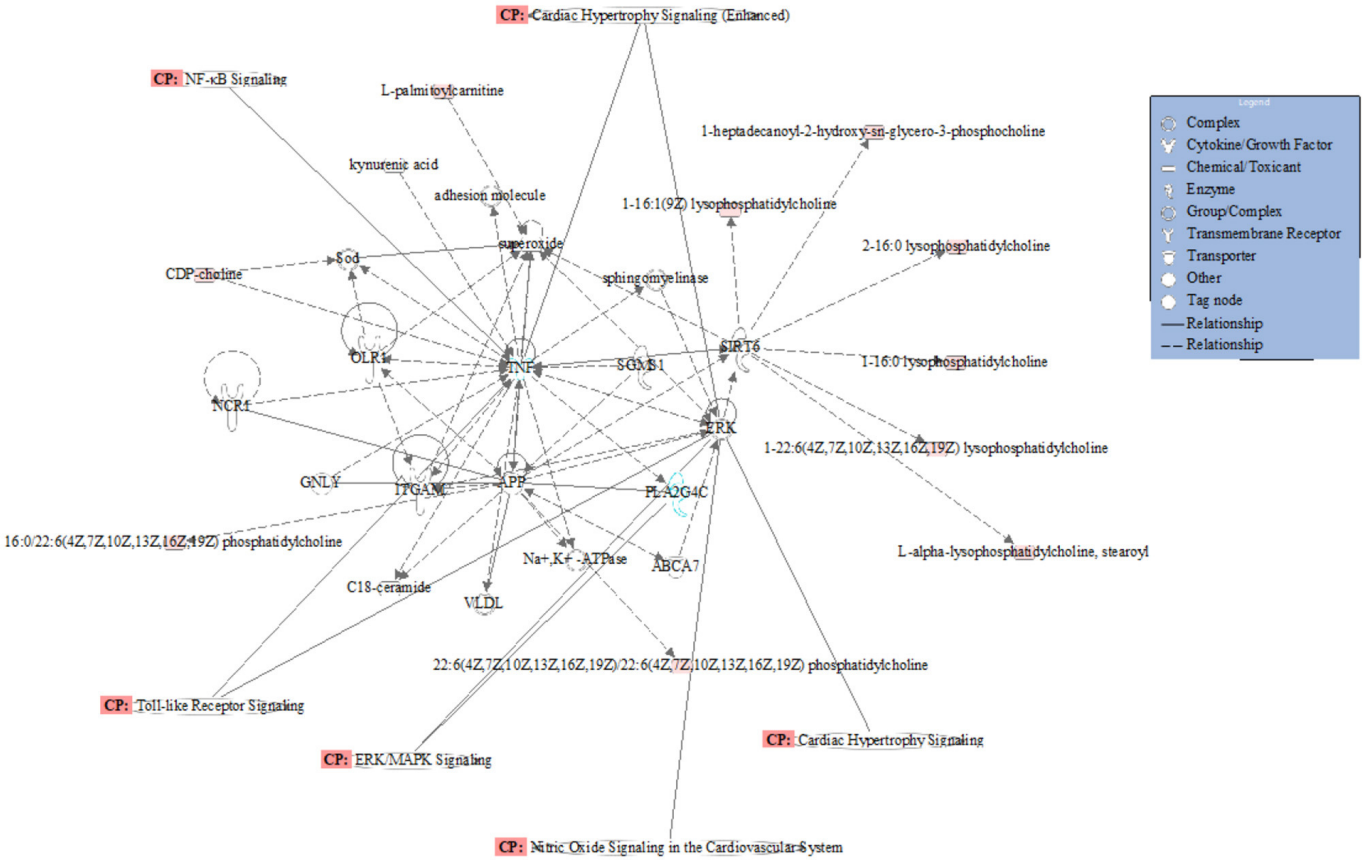
Network and function analysis of differential metabolites using IPA database. The red nodes represent the upregulated metabolites. Green nodes represent the downregulated metabolites. CP represents the signaling pathway related to the changed metabolites.

A total of 35 biomarkers were upregulated, and 11 were downregulated in the ACS group. The main classes of the biomarkers were lyso-phosphatidylcholine (LPC), primary amides, and lsoSMs, as shown in Figures 5A,B. Metaboanalyst and KEGG databases were used for biomarker pathway enrichment analysis. As shown in Figures 5C,D, these markers were mainly enriched in four major metabolic pathways, including phospholipid biosynthesis, alpha linolenic acid and linoleic acid metabolism, homocysteine degradation, and plasmalogen synthesis. IPA network analysis showed that these biomarkers were related to the cardiac hypertrophy signaling, ERK/MAPK signaling pathway, NF-kappa B signaling pathway, nitric oxide (NO) signaling pathway in cardiovascular system, and TLR-signaling pathway, as shown in Figure 6.

## Discussion

CHD is a myocardial ischemia and hypoxia disease caused by coronary artery plaque forming coronary stenosis. Its incidence rate is increasing every year. Common risk factors include age, sex, obesity, diabetes, hypertension, smoking, etc. In recent years, with unhealthy diet and high life pressure, the age of onset tends to be lower. Currently, the latest cardiovascular guidelines (ESC 2019) (19) for the diagnosis and management of chronic coronary syndromes proposed that CHD can be divided into chronic coronary syndrome and ACSs. From a pathophysiological point of view, ACSs is an acute thrombosis after the rupture of unstable plaque, which leads to acute myocardial ischemia, which can further cause acute heart failure, malignant arrhythmia, and even sudden death. At present, the gold standard for the diagnosis of coronary artery stenosis is coronary angiography. Coronary angiography is a minimally invasive procedure, which is expensive and may cause many complications, such as bleeding at the puncture site, anesthetic allergy, iodine contrast agent allergy, contrast nephropathy, gastrointestinal bleeding, and cerebral hemorrhage. A cheaper and safer diagnostic method is needed for patients and doctors. Metabolomics studies of the metabolites closely related to the disease provide a better idea regarding the diagnosis, prognosis, and mechanism of the disease. Our study is an untargeted metabolomics study of serum potential biomarkers in ACS patients based on UPLC-Orbitrap/MS. In total, 69 biomarkers were enriched in 19 metabolic pathways, 43 biomarkers were increased significantly, and 26 biomarkers were down-regulated significantly. The main classes were lyso-SM, cinnamic acids, cholines, and primary amides. In these metabolic pathways, fatty acid metabolism and phospholipid metabolism were found to be closely related to ACS.

PS (phosphatidylserine) is a kind of phospholipid with procoagulant activity (20, 21). PS is released from blood cells (such as platelets, white blood cells, and red blood cells) and vascular wall cells (such as endothelial cells and smooth muscle cells) when they are stimulated by vascular high stress and oxidation products (22). PS can then activate coagulation factors and promote thrombosis. The PS content was positively correlated with the severity of the ACSs (23). In our study, 13 PS were increased in the ACS group, such as PS (15:0/22:0), PS (15:0/24:0), PS (22:2 (13z, 16Z)/15:0), and PS (15:0/24:1 (15z)), which were significantly higher than those in the control group, and their levels were positively correlated with the severity of the disease. These metabolites may be released from myocardial cells and participate in the formation of thrombosis stimulated by a high vascular stress and oxidation products.

In addition, lysophosphatide was another main altered phospholipid in our study. It can be divided into lysophosphatidic acid (LysoPA), sphingosine 1-phosphate (S1P), lysophosphatidyl inositol (LysoPI), lysophosphatidyl serine (LysoPS), and lysophosphatidylthanolamine (LysoPE). They are bioactive lipids that participate in physiological and pathophysiological processes through their specific cell surface receptors. Moreover, these different kinds of metabolites have different functions in coronary artery and peripheral blood vessels in ACS. For example, lysoPI could predict the prognosis of ACS after percutaneous coronary intervention in a previous study (24, 25). In our study, the lysoPI (0:0/18:0) and lysoPI(20:4 (5Z, 8Z, 11z, 14z)/0:0) ACS groups were significantly increased in the ACS group.

Lysophosphatidylcholine (LysoPCs) is a proinflammatory factor that leads to atherosclerosis (26). It is an important component of oxidized low-density lipoprotein (ox-LDL) and has a wide range of biological effects (27). LysoPCs induce inflammation, activate macrophages and T lymphocytes, and change vascular smooth muscle cells (28). LysoPCs can also activate intracellular PKC, promote the expression of VCAM-1 and ICAM-1, increase the production of oxygen free radicals, inhibit the release of NO from endothelial cells, and play multiple roles in the inflammatory response and remodeling of various cells in the vascular wall (29). Early studies have shown that lysoPCs can induce apoptosis in human coronary artery smooth muscle cells and that their content changes are closely related to ACS. On the one hand, lysoPCs can be used as a marker of cardiac injury; on the other hand, drugs to reduce the lysoPCs level can be used to alleviate a series of inflammatory reactions caused by lysoPCs. In our study, lysoPC(20:4(8Z,11Z,14Z,17Z)/0:0), lysoPC(p-18:0/0:0), lysoPC (p-16:0/0:0), and lysoPC(18:0) were significantly increased. And lysoPC(20:4(8Z,11Z,14Z,17Z)/0:0) showed a high ACS diagnostic ability with an AUC value of 0.936. In a word, these phospholipids that are significantly changed in patients with ACS can not only be used as diagnostic markers of ACS but also some of them may be developed into drugs to treat ACS.

Compared with the control group, the serum linoleic acid content in ACS patients increased. High blood levels of linoleic acid, but low levels of trans oleic acid, are inversely associated with ACS (30). L-carnitine is an essential auxiliary factor in fatty acid B oxidation (31). It plays an important role in promoting aerobic metabolism and eliminating toxic substances in energy metabolism. It is especially important for tissues with a high energy consumption, such as the myocardium and skeletal muscle (32). The contents of L-carnitine and adenosine triphosphate (ATP) in the ischemic myocardium decreased significantly, and the free fatty acids increased, which led to the energy metabolism disorder of heart cells (33). The level of L-carnitine in the myocardium decreased significantly, but its level in the blood increased. It is speculated that myocardial ischemia may lead to the release of part of L-carnitine into human blood or that the transport of L-carnitine into cardiomyocytes may be blocked, resulting in an increase in the plasma. In our study, stearic acid, linoleic acid, and oleic acid were significantly increased. This may be related to the accumulation of fatty acids caused by energy metabolism damage after myocardial injury. In conclusion, the oxidation of fatty acids must be completed in mitochondria with the help of carnitine, which is an important source of energy. The abnormal changes of carnitine and fatty acids in the patients of ACS suggest that the abnormality of energy metabolism in the ACS patients may be related to mitochondrial dysfunction. In the future, we can develop new drugs to improve mitochondrial function to treat ACS.

## Conclusion

We performed a metabonomics study on ACS serum samples based on UPLC-Orbitrap/MS system. The key changed metabolites and metabolic pathways of ACS were identified. Some of them showed high diagnostic ability for ACS. Signaling pathways related to the pathogenesis of ACS have also been found, which provides a reference for us to study the relationship between metabolites and the pathogenesis of ACS. Our findings will provide a demonstration for the study of diagnostic markers and pathogenesis of ACS.

## Data Availability Statement

The datasets presented in this study can be found in online repositories. The names of the repository/repositories and accession number(s) can be found below: EBI MetaboLights database [accession: MTBLS2482].

## Ethics Statement

The studies involving human participants were reviewed and approved by Committee of Shanghai Tongren Hospital. The patients/participants provided their written informed consent to participate in this study. Written informed consent was obtained from the individual(s) for the publication of any potentially identifiable images or data included in this article.

## Author Contributions

TJ and ZQ conceived and designed the experiments. LS performed the experiments. ZZ analyzed the data. LS and ZZ wrote the paper. All authors read and approved the final manuscript.

## Conflict of Interest

The authors declare that the research was conducted in the absence of any commercial or financial relationships that could be construed as a potential conflict of interest.
