# Supplementary material for: Serum Metabonomic Study of Patients With Acute Coronary Syndrome Using Ultra-Performance Liquid Chromatography Orbitrap Mass Spectrometer
Source: Front Cardiovasc Med. 2021 Feb 26;8:637621. doi: 10.3389/fcvm.2021.637621 (PMC7953136; doi:10.3389/fcvm.2021.637621)
Supplement: Supplementary file 1 [file Table_1.docx]

Supplemental Material

Serum metabonomic study of patients with acute coronary syndrome using ultra performance liquid chromatography orbitrap mass spectrometer

Lei Song^1,2 #^, Zhongxiao Zhang^3,#^, Zhaohui Qiu^2,*^,Tingbo Jiang^1,*^

1. The First Affiliated Hospital of Soochow University, Suzhou 215006, China.
2. Tongren Hospital, Shanghai Jiao Tong University School of Medicine, 1111 XianXia Road, Shanghai 200336, China.
3. Hongqiao International Institute of Medicine, Tongren Hospital, Shanghai Jiao Tong University School of Medicine, 720 XianXia Road, Shanghai 200336, China.

***.** Corresponding author: Tingbo Jiang, The First Affiliated Hospital of Soochow University, Suzhou 215006, China.

E-mail address: 18906201122@189.cn.

Zhaohui Qiu, Tongren Hospital, Shanghai Jiao Tong University School of Medicine, 1111 XianXia Road, Shanghai 200336, China. E-mail address: QZH3503@shtrhospital.com.

# These authors contributed equally to this work.

Supplemental MaterialTable S1.The changed metabolites in the serum of ACS patients compared with the control group.

| M/Z | VIP | Name | HMDB | RT [min] | FC(ACS/Control) | FDR | p |
| --- | --- | --- | --- | --- | --- | --- | --- |
| 805.58888 | 2.2 | PS(15:0/22:0) | HMDB0112334 | 8.1 | 28.2 | 5.0E-12 | 5.8E-13 |
| 600.32847 | 2.4 | LysoPI(0:0/18:0) | HMDB0061704 | 8.1 | 16.0 | 6.8E-17 | 5.0E-18 |
| 750.55183 | 2.6 | PG(a-13:0/a-21:0) | HMDB0116640 | 8.1 | 10.6 | 2.1E-18 | 1.2E-19 |
| 858.69527 | 2.5 | PA(22:0/a-25:0) | HMDB0115667 | 8.1 | 11.4 | 1.4E-18 | 4.0E-20 |
| 833.61952 | 2.5 | PS(15:0/24:0) | HMDB0112340 | 8.1 | 8.9 | 4.8E-16 | 4.2E-17 |
| 801.56107 | 2.4 | PS(22:2(13Z,16Z)/15:0) | HMDB0112763 | 8.3 | 14.4 | 1.0E-18 | 1.5E-20 |
| 767.55716 | 1.8 | PE-NMe(15:0/22:4(7Z,10Z,13Z,16Z)) | HMDB0113034 | 12.4 | 10.3 | 3.8E-09 | 7.9E-10 |
| 763.50734 | 1.3 | PE(16:0/22:6(4Z,7Z,10Z,13Z,16Z,19Z)) | HMDB0008946 | 11.0 | 5.4 | 8.3E-04 | 5.3E-04 |
| 527.30092 | 1.7 | LysoPE(0:0/22:5(4Z,7Z,10Z,13Z,16Z)) | HMDB0011494 | 7.7 | 6.0 | 1.7E-06 | 6.1E-07 |
| 674.53517 | 1.6 | SM(d18:1/14:0) | HMDB0012097 | 9.6 | 2.9 | 4.5E-03 | 3.6E-03 |
| 862.56965 | 1.9 | PI(16:0/20:2(11Z,14Z)) | HMDB0009786 | 12.4 | 4.0 | 1.3E-11 | 1.7E-12 |
| 729.5293 | 1.5 | PC(14:0/18:2(9Z,12Z)) | HMDB0007874 | 17.0 | 2.6 | 5.0E-03 | 4.0E-03 |
| 765.52213 | 1.6 | PC(15:0/20:5(5Z,8Z,11Z,14Z,17Z)) | HMDB0007951 | 10.5 | 2.4 | 4.7E-05 | 2.4E-05 |
| 723.51244 | 1.7 | PE(18:3(6Z,9Z,12Z)/P-18:1(11Z)) | HMDB0009149 | 14.8 | 2.6 | 4.0E-06 | 1.6E-06 |
| 541.31694 | 1.3 | LysoPC(20:5) | HMDB0010397 | 8.2 | 10.4 | 1.4E-11 | 2.1E-12 |
| 495.33204 | 1.8 | LysoPC(16:0/0:0) | HMDB0010382 | 9.1 | 4.3 | 2.2E-15 | 2.3E-16 |
| 775.56203 | 1.4 | PE(22:5(4Z,7Z,10Z,13Z,16Z)/P-18:1(11Z)) | HMDB0009644 | 10.3 | 2.8 | 3.0E-05 | 1.4E-05 |
| 851.5565 | 1.5 | PE-NMe(22:5(4Z,7Z,10Z,13Z,16Z)/22:6(4Z,7Z,10Z,13Z,16Z,19Z)) | HMDB0113643 | 17.0 | 2.4 | 1.1E-04 | 6.0E-05 |
| 887.57442 | 1.6 | PS(22:2(13Z,16Z)/22:6(4Z,7Z,10Z,13Z,16Z,19Z)) | HMDB0112785 | 12.4 | 2.8 | 7.2E-07 | 2.2E-07 |
| 533.3488 | 1.4 | LysoPE(0:0/22:2(13Z,16Z)) | HMDB0011492 | 9.2 | 4.4 | 2.4E-09 | 4.2E-10 |
| 779.57838 | 1.6 | PE(22:4(7Z,10Z,13Z,16Z)/P-18:0) | HMDB0009610 | 12.4 | 2.6 | 1.1E-06 | 3.8E-07 |
| 620.2976 | 1.3 | LysoPI(20:4(5Z,8Z,11Z,14Z)/0:0) | HMDB0061690 | 11.2 | 3.4 | 5.0E-07 | 1.5E-07 |
| 537.51268 | 1.7 | N-Palmitoylsphingosine | HMDB0000790 | 15.4 | 2.8 | 1.5E-09 | 2.4E-10 |
| 777.56184 | 1.2 | PE(22:4(7Z,10Z,13Z,16Z)/P-18:1(11Z)) | HMDB0009611 | 10.2 | 2.1 | 1.0E-03 | 6.9E-04 |
| 748.52754 | 1.2 | PG(16:0/18:1(11Z)) | HMDB0010573 | 12.4 | 3.0 | 3.3E-06 | 1.3E-06 |
| 804.5713 | 1.7 | PA(20:5(5Z,8Z,11Z,14Z,17Z)/24:1(15Z)) | HMDB0115223 | 6.0 | 1.9 | 2.3E-05 | 1.0E-05 |
| 739.52308 | 1.4 | PE-NMe(15:0/20:4(5Z,8Z,11Z,14Z)) | HMDB0113028 | 14.5 | 1.8 | 5.7E-04 | 3.5E-04 |
| 759.57091 | 1.4 | PC(14:0/20:1(11Z)) | HMDB0007879 | 11.4 | 2.6 | 1.3E-04 | 6.9E-05 |
| 773.5339 | 1.9 | PE(22:6(4Z,7Z,10Z,13Z,16Z,19Z)/P-18:1(11Z)) | HMDB0009710 | 17.0 | 1.7 | 1.1E-03 | 7.9E-04 |
| 827.54298 | 1.8 | PC(18:3(6Z,9Z,12Z)/22:6(4Z,7Z,10Z,13Z,16Z,19Z)) | HMDB0008189 | 16.9 | 1.9 | 3.0E-04 | 1.7E-04 |
| 895.54465 | 1.1 | PS(DiMe(11,3)/DiMe(11,5)) | HMDB0061553 | 14.9 | 1.5 | 2.5E-02 | 2.2E-02 |
| 284.26881 | 1.4 | Stearic acid | HMDB0000827 | 9.6 | 2.2 | 3.5E-09 | 6.6E-10 |
| 803.54166 | 1.4 | PC(16:1(9Z)/22:6(4Z,7Z,10Z,13Z,16Z,19Z)) | HMDB0008023 | 12.5 | 1.6 | 1.5E-02 | 1.3E-02 |
| 507.3675 | 2.4 | LysoPC(P-18:0/0:0) | HMDB0013122 | 10.7 | 1.7 | 1.2E-08 | 2.7E-09 |
| 765.53739 | 1.2 | PE-NMe(15:0/22:5(7Z,10Z,13Z,16Z,19Z)) | HMDB0112978 | 9.8 | 1.7 | 1.3E-04 | 7.1E-05 |
| 313.2252 | 1.3 | 9-Decenoylcarnitine | HMDB0013205 | 4.8 | 1.7 | 5.7E-04 | 3.6E-04 |
| 777.52862 | 1.2 | PC(14:0/22:6(4Z,7Z,10Z,13Z,16Z,19Z)) | HMDB0007892 | 16.9 | 1.4 | 5.6E-02 | 5.5E-02 |
| 779.5429 | 1.3 | PC(14:0/22:5(4Z,7Z,10Z,13Z,16Z)) | HMDB0007890 | 9.6 | 1.4 | 2.6E-02 | 2.4E-02 |
| 830.57674 | 1.2 | PA(22:6(4Z,7Z,10Z,13Z,16Z,19Z)/24:1(15Z)) | HMDB0115427 | 14.8 | 1.4 | 1.2E-03 | 8.7E-04 |
| 479.33639 | 1.9 | LysoPC(P-16:0/0:0) | HMDB0010407 | 8.9 | 1.5 | 1.1E-06 | 3.4E-07 |
| 525.36723 | 1.6 | Cytidine 5'-diphosphocholine | HMDB0001413 | 10.0 | 1.5 | 2.2E-06 | 8.1E-07 |
| 425.35025 | 1.2 | Oleoylcarnitine | HMDB0005065 | 7.5 | 1.4 | 3.0E-03 | 2.2E-03 |
| 399.33468 | 1.3 | Palmitoylcarnitine | HMDB0000222 | 7.4 | 1.4 | 3.5E-04 | 2.1E-04 |
| 686.48928 | 1.1 | PA(15:0/20:2(11Z,14Z)) | HMDB0115508 | 16.9 | 0.8 | 1.3E-02 | 1.1E-02 |
| 743.54698 | 1.3 | PE(18:1(11Z)/18:1(11Z)) | HMDB0009025 | 12.6 | 0.8 | 2.3E-02 | 2.0E-02 |
| 644.44267 | 1.1 | PA(14:0/18:2(9Z,12Z)) | HMDB0114779 | 10.6 | 0.8 | 3.3E-02 | 3.2E-02 |
| 1378.94915 | 1.1 | CL(16:0/16:0/16:0/18:1(11Z)) | HMDB0056390 | 7.9 | 0.7 | 1.2E-02 | 1.0E-02 |
| 548.04466 | 1.1 | UDP-4-dehydro-6-deoxy-D-glucose | HMDB0012300 | 8.4 | 0.7 | 7.5E-03 | 6.2E-03 |
| 1334.9222 | 1.0 | CL(i-14:0/i-12:0/18:2(9Z,11Z)/i-19:0) | HMDB0075769 | 8.0 | 0.7 | 2.9E-02 | 2.8E-02 |
| 1422.98093 | 1.1 | CL(16:0/16:0/16:1(9Z)/22:6(4Z,7Z,10Z,13Z,16Z,19Z)) | HMDB0056416 | 7.8 | 0.7 | 2.9E-02 | 2.8E-02 |
| 724.51903 | 1.3 | PA(16:0/22:4(7Z,10Z,13Z,16Z)) | HMDB0114846 | 10.6 | 0.7 | 4.4E-03 | 3.5E-03 |
| 817.59524 | 1.1 | PC(22:5(4Z,7Z,10Z,13Z,16Z)/P-18:1(11Z)) | HMDB0008687 | 11.4 | 0.9 | 5.8E-01 | 5.8E-01 |
| 1390.98716 | 1.4 | CL(i-13:0/a-21:0/18:2(9Z,11Z)/i-15:0)[rac] | HMDB0073037 | 10.2 | 0.7 | 3.1E-03 | 2.4E-03 |
| 813.62847 | 1.2 | PE-NMe(16:1(9Z)/24:1(15Z)) | HMDB0113083 | 17.0 | 0.6 | 1.7E-03 | 1.2E-03 |
| 791.54732 | 1.1 | PE-NMe2(16:0/22:6(4Z,7Z,10Z,13Z,16Z,19Z)) | HMDB0113959 | 12.2 | 0.7 | 2.8E-03 | 2.0E-03 |
| 747.51995 | 1.6 | PE(P-16:0/22:6(4Z,7Z,10Z,13Z,16Z,19Z)) | HMDB0005780 | 17.0 | 0.7 | 1.1E-03 | 7.2E-04 |
| 781.56182 | 1.3 | PC(14:0/22:4(7Z,10Z,13Z,16Z)) | HMDB0007889 | 8.1 | 0.5 | 3.3E-04 | 1.9E-04 |
| 801.52806 | 1.3 | PC(18:3(6Z,9Z,12Z)/20:5(5Z,8Z,11Z,14Z,17Z)) | HMDB0008182 | 12.5 | 0.7 | 2.9E-02 | 2.7E-02 |
| 803.55696 | 1.3 | PE-NMe(18:1(11Z)/22:6(4Z,7Z,10Z,13Z,16Z,19Z)) | HMDB0113134 | 8.9 | 0.4 | 1.9E-07 | 5.2E-08 |
| 879.59969 | 1.4 | PE-NMe(11D3/13M5) | HMDB0113761 | 8.6 | 0.5 | 6.4E-06 | 2.6E-06 |
| 844.65775 | 1.2 | TG(18:4(6Z,9Z,12Z,15Z)/16:1(9Z)/18:4(6Z,9Z,12Z,15Z)) | HMDB0055405 | 9.9 | 0.4 | 3.8E-05 | 1.9E-05 |
| 846.68233 | 1.7 | TG(14:0/18:3(6Z,9Z,12Z)/20:5(5Z,8Z,11Z,14Z,17Z)) | HMDB0042567 | 8.3 | 0.5 | 2.6E-05 | 1.2E-05 |
| 747.61235 | 2.0 | PC(O-16:0/18:0) | HMDB0013405 | 12.2 | 0.4 | 1.2E-08 | 2.8E-09 |
| 834.60755 | 1.7 | PA(22:4(7Z,10Z,13Z,16Z)/24:1(15Z)) | HMDB0115348 | 8.2 | 0.4 | 1.2E-07 | 3.1E-08 |
| 832.59328 | 1.7 | PA(22:5(4Z,7Z,10Z,13Z,16Z)/24:1(15Z)) | HMDB0115375 | 8.3 | 0.4 | 1.6E-05 | 6.6E-06 |
| 704.58224 | 2.0 | SM(d18:0/16:0) | HMDB0010168 | 17.0 | 0.3 | 3.8E-05 | 1.9E-05 |
| 773.62987 | 2.7 | PC(o-16:1(9Z)/20:0) | HMDB0013414 | 9.9 | 0.2 | 2.0E-18 | 9.0E-20 |
| 832.6653 | 1.4 | TG(15:0/18:4(6Z,9Z,12Z,15Z)/18:4(6Z,9Z,12Z,15Z)) | HMDB0043679 | 9.4 | 0.2 | 2.8E-08 | 6.9E-09 |
